# Bulk and Interfacial Behavior of Potato Protein-Based Microgels

**DOI:** 10.1021/acs.langmuir.4c01785

**Published:** 2024-10-01

**Authors:** Daisy Z. Akgonullu, Nicholas M. O’Hagan, Brent S. Murray, Simon D. Connell, Yuan Fang, Bruce R. Linter, Anwesha Sarkar

**Affiliations:** † Food Colloids and Bioprocessing Group, School of Food Science and Nutrition, University of Leeds, Leeds LS2 9JT, U.K.; ‡ Molecular and Nanoscale Physics Group, School of Physics and Astronomy, University of Leeds, Leeds LS2 9JT, U.K.; § PepsiCo, Valhalla, New York, New York 10595, United States; ∥ PepsiCo International Ltd., Leicester LE4 1ET, U.K.

## Abstract

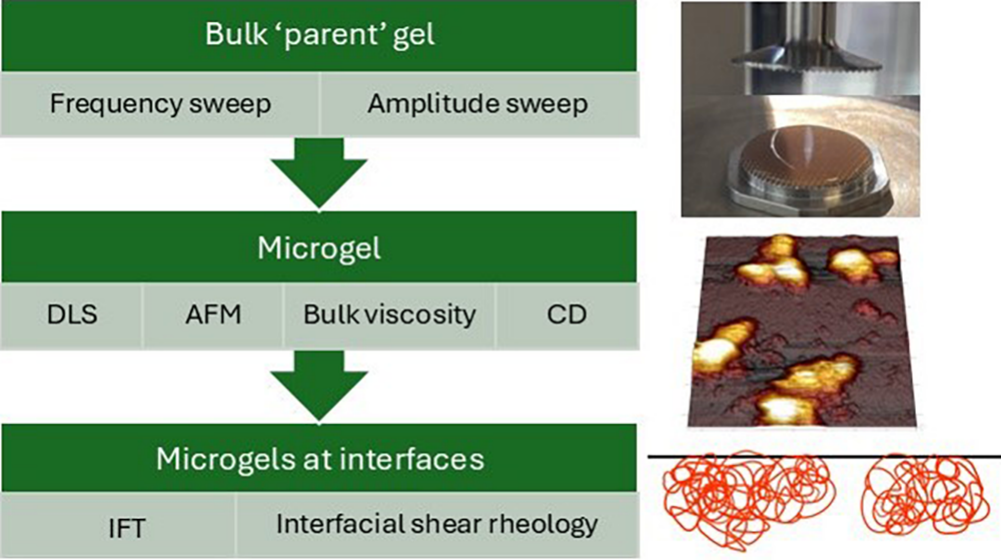

This study aims to understand the bulk and interfacial performance of potato protein microgels. Potato protein (PoP) was used to produce microgels of submicrometer diameter via a top-down approach of thermal cross-linking followed by high-shear homogenization of the bulk gel. Bulk “parent” gels were formed at protein concentrations [PoP] = 5–18 wt %, which subsequently varied in their bulk shear elastic modulus (*G*′) by several orders of magnitude (1–100 kPa), *G*′ increasing with increasing [PoP]. The PoP microgels (PoPM) formed from these parent gels had diameters varying between 100 and 300 nm (size increasing with increasing *G*′ and [PoP]), as observed via dynamic light scattering and atomic force microscopy (AFM) of PoPM adsorbed onto silicon. Interfacial rheology (interfacial shear storage and loss moduli, *G*_*i*_′ and *G*_*i*_″) and interfacial tension (γ) of adsorbed films of PoP (i.e., nonheated PoP) and PoPM (both at tetradecane–water interfaces) were also studied, as well as the bulk rheology of the PoPM dispersions. The results showed that PoPM dispersions (at 50 vol %) had significantly higher bulk viscosity and shear thinning properties compared to the nonmicrogelled PoP at the same overall [PoP], but the bulk rheological behavior was in sharp contrast to the interfacial rheological performance, where *G*_*i*_′ and *G*_*i*_″ of PoP were higher than for any of the PoPM. This suggests that the deformability and size of the microgels were key in determining the interfacial rheology of the PoPM. These findings may be attributed to the limited capacity for “unfolding” and lateral interactions of the larger PoPM at the interface, which are presumed to be stiffer due to their production from the strongest PoP gels. Our study further confirmed that heating and cooling the adsorbed films of PoPM after their adsorption showed little change, highlighting that hydrogen bonding was limited between the microgel particles.

## Introduction

The search for new emulsifiers that meet the criteria of environmentally friendly, biocompatible, and food-grade is a topical research area.^1^ Demand is moving from common oil-based, low-molecular-weight (*M*_w_) stabilizers^2^ such as polyglycerol polyricinoleate (PGPR), polysorbates, and mono- and di- glycerides to more clean-label protein-based alternatives.^3^ Emulsions stabilized by dairy protein, e.g., whey protein, caseins, and their derivatives are well studied and widely used in industry.^4,5^ They exhibit desirable interfacial stability through their ability to reduce interfacial tension and/or form strong viscoelastic layers; these are important to promote electrostatic and steric repulsion to prevent droplet aggregation and coalescence.^1,6^ However, the need for emulsifiers originating from more renewable and environmentally friendly sources is increasingly apparent,^2,7^ as a transition toward plant protein is imperative in achieving “net zero” emissions targets.^8^ More specifically, interest in stabilizers of plant protein origin is at the forefront of development for environmental sustainability,^2,7^ but a thorough investigation of their functionality is still required^2,9^ for these plant-based emulsifiers to successfully replace current industry standards as effective stabilizers of emulsions and foams.

Besides plant proteins being subject to natural variations in their structure, their adoption as colloid stabilizers poses further difficulties due to their low solubility, rigidity,^10^ and aggregation.^11^ When used at interfaces, in general, plant proteins have been reported to yield weaker interfacial layers than proteins of animal origin.^2^ Optimization of protein structure and extraction methods are recommended to allow for physiochemical modification of plant proteins under milder conditions,^2^ but the specificity in these methods goes hand in hand with a need for further understanding of their protein structure.^3^ Recently, microgels have emerged as a means of utilizing biopolymers in a less specific way to create soft particles that might fulfill the role of such stabilizers. Each microgel particle is composed of a mesh-like structure of hydrated polymer.^12^ Microgels produced from a range of biopolymers have been studied within bulk solution and at interfaces but most frequently reported are those formed from whey protein.^13^ Increasingly studies are starting to explore microgels originating from polysaccharides e.g., pectin,^14^ chitosan,^15^ and plant proteins, e.g., soy protein,^16^ pea protein,^17^ and potato protein (PoP).^18^

Microgels have the ability to modify the viscoelasticity of bulk media, which has been observed in both dairy^12^ and plant-derived samples.^18^ At high bulk concentrations, microgels have been suggested to form an interconnected network across the dispersion, forming an elastic solid^19^ that may also aid emulsion stability via immobilization of the droplets within this network, sometimes also with the droplets themselves flocculating with this network.^14^ It has been proposed that microgels can give a much larger range of viscosity control than systems of rigid particles, since their deformable structure allows actual interpenetration of neighboring particles at high particle density.^13^ This allows for an even wider range of effective particle volume fractions than for hard spheres and promotes shear dependence within the system.^20^

Microgels may thus act to promote emulsion stability via both bulk and interfacial mechanisms: research has clearly demonstrated their ability to act as Pickering-like stabilizers.^17,21^ Biopolymeric microgels have been observed to adsorb to interfaces with high desorption energy and provide a significant steric barrier to emulsion droplets to prevent destabilization.^14^ However, variations in the effectiveness of different microgel systems are still widely debated. For example, the elasticity of microgel particles is often cited as a determining factor in their stabilizing efficiency,^13^ since their capacity to compress and adapt their structure may provide microgels with greater resilience to fluctuations in their environment and avoid destabilization.^22^

Potato is a sustainable source of protein, which displays ease of denaturation^23,24^ and solubility.^25−27^ These attributes are due to potato protein’s lack of internal disulfide bonding, which enables greater protein unfolding and exposure of interior hydrophobic amino acid residues.^28^ Such residues facilitate the creation of hydrophobic bonds between adjacent molecules^26^ that may explain the lower concentration of potato protein required for gel formation, compared to other protein sources.^23,29^ Additionally, potato protein poses the potential to be obtained from food industry waste streams,^23^ offering sourcing opportunities to support a circular economy. Potato protein has been shown to be an effective emulsion stabilizer,^30^ while the various components of the protein contribute differently to this stability.^31^ Potato protein is comprised of three main fractions: patatin, protease inhibitors, and high *M*_w_ proteins.^32^ It has been frequently noted that the exact composition of potato protein is highly variable—depending on the extraction method, time of harvest, and cultivar.^32^ The most prevalent fraction is patatin, representing 40–60% of the total protein,^32^ and most studies report work on patatin-rich commercial potato protein.^24^

Potato protein bulk (macro-) gel formation and properties have been extensively studied,^23,24,26,33,34^ which has led to findings of its unique responsiveness to variations in environmental stimuli (e.g., temperature, pH, and ionic strength) when compared to whey protein gels.^26,33^ This behavior is associated with observations of low levels of covalent bonding and a high tendency to form dense aggregates. When converted into microgels, potato protein has shown its excellent potential to act as a viscosity modifier and lubricant for food applications.^18^ Microgelled potato protein has also been proposed to improve the capacity of potato protein as an emulsion stabilizer.^35,36^

In the literature, milk proteins, particularly β-lactoglobulin, have been widely studied via interfacial rheology, leading to this protein being considered a benchmark in terms of its excellent stabilizing ability.^2^ Its relatively small structure and ease of unfolding provide the protein with flexibility at the interface.^6^ However, as they become unfolded, the β-lactoglobulin molecules adopt comparatively thin interfacial layers, which reduces their steric stabilizing effect.^1,6,37^ The formation of thick films around emulsion droplets is generally more effective in providing more long-term steric stabilization against coalescence and Ostwald ripening.^7^ This is why protein microgels, due to their larger size and dense gelled structure, may be more effective, acting partly like classic hard particle Pickering stabilizers but also unfolding and cross-linking at the interface like massive globular proteins.^25^

Despite interfacial characterization of adsorbed potato protein becoming more widely researched via Langmuir trough isotherms,^30,38^ surface shear,^31^ and surface dilatational measurements,^30,31,38,39^ the mechanisms dictating the “strength” of adsorbed potato protein films and its relationship to emulsion stability are still largely unknown.^30,31^ Moreover, to the authors’ knowledge, there has been no investigation of potato protein-based microgels using any of these means of interfacial characterization. Therefore, this study aims to investigate the role of potato protein concentration in determining the softness and size of the corresponding microgels and the influence of these factors on bulk and interfacial behavior in order to optimize their applications in food emulsions and oral tribology.

## Experimental Section

### Materials

Sosa “Potatowhip” potato protein, containing ∼90% protein, was purchased from Henley Bridge (Lewes, U.K.). Previous work from Kew et al.^27^ has confirmed that this sample is mainly formed of patatin; therefore, further discussion of the protein is made with patatin as a reference. Tetradecane and 4-(2-hydroxyethyl)-1-piperazineethanesulfonic acid (HEPES) buffer were obtained from Fisher Scientific UK Ltd. (Loughborough, U.K.). Silicon wafers of type 100 were obtained from Agar Scientific Ltd. (Essex, U.K.), and all atomic force microscopy (AFM) cantilevers were sourced from Bruker UK Ltd. (Coventry, U.K.). All other chemicals were purchased from Fisher Scientific UK Ltd. (Loughborough, U.K.), and all solutions were prepared with Milli-Q water (purified using Milli-Q apparatus, Millipore Corp., Bedford, MA).

### Preparation of Potato Protein Solutions

A solution of 20 mM HEPES at pH 7.0 was used as a buffer for all dispersions. Potato protein solutions (PoPS) were prepared at varying concentrations (5, 10, 15, and 18 wt %, the latter chosen as the maximum potato protein content found to be soluble in solution) and stirred at room temperature for a minimum of 2 h to ensure complete dissolution of the protein. Calculations of the final protein concentrations were based on the actual protein concentration of the powder (∼90%). Sodium azide (0.02 wt %) was added to the samples for bacteriostatic preservation.

### Preparation of Potato Protein Microgels

Microgel fabrication was based on the previous methodology of Sarkar et al.,^40^ Soltanahmadi et al.,^41^ and Aery et al.^36^ PoPS were heated in a water bath at 80 °C for 30 min, followed by cooling in room temperature water for 10 min and refrigeration overnight at 4 °C. This gelled the protein, and the gel was then diluted at 1:1 w/w ratio with HEPES buffer at pH 7.0 and sheared for 3 min at 12,500 rpm using a hand blender (Bosch MSM6B150GB, U.K.). The dispersion of gel fragments formed was degassed (Intertronics, Thinky ARE-250), followed by 1 min of mixing at 2000 rpm and 1 min of defoaming at 2200 rpm. Samples were finally passed through a custom-made jet homogenizer (Jet Homogenizer, University of Leeds, U.K.) for 3 cycles at 300 bar. Considering the bulk “parent” gels as 100 vol %, post 1:1 dilution, the resultant potato protein microgel dispersions can be thought of as a composition of 50 vol % microgels. These dispersions are subsequently referred to as PoPM-*X*, with the “*X*” denoting the wt % PoPS from which the “parent” gel was formed: see Table 1.

**Table 1:** Summary of Samples Tested

sample	abbreviation
potato protein solution	PoPS
potato protein microgel containing 5 wt % protein	PoPM-5
potato protein microgel containing 10 wt % protein	PoPM-10
potato protein microgel containing 15 wt % protein	PoPM-15
potato protein microgel containing 18 wt % protein	PoPM-18

### Rheology of Parent Gels

PoPS at 5–18 wt % were prepared as described above. PoPS were then added to a serrated parallel plate geometry (PP25/P2, diameter: 25 mm) at 1 a mm gap in a controlled stress rheometer (MCR-302, Anton Paar, Austria) and sealed with silicone oil (350 cSt) as a solvent trap. The PoPS was subjected to a temperature ramp of 25–80 °C at a rate of 0.08 °C s^–1^ to form a macrogel in situ in the rheometer. Once at 80 °C, the gels were held at a constant temperature for 10 min before being cooled from 80 to 25 °C, at which point the gels were subject to oscillatory shear rheology tests. (Although these conditions generate a slightly different time–temperature profile to those gels that were subsequently broken down to form PoPM, we still refer to them here as “parent” gels.) Oscillatory strain amplitude sweeps were conducted at a constant angular frequency of 6.283 rad s^–1^ (1 Hz) from 0.01 to 100% strain. Oscillatory frequency sweeps were measured at a constant strain of 0.1% for an angular frequency of 0.1 to 100 rad s^–1^.

### Dynamic Light Scattering (DLS)

The particle size distributions of PoPS and PoPM were determined at 25 °C using dynamic light scattering via a Zetasizer Nano-ZS (Malvern Instruments Ltd., Malvern, Worcestershire, U.K.). Samples were diluted to 0.01 vol % for PoPM and 0.01 wt % for PoPS and added to standard disposable cuvettes. The refractive index of the potato protein-based samples was set to 1.45 with an absorption of 0.001.

### Atomic Force Microscopy (AFM)

Dispersions of PoPM were diluted to a protein concentration of 0.01 wt %, and approximately 150 μL of diluted sample was deposited onto new, clean silicon wafers. Samples were then left for 10 min to adsorb to the surface and then “washed” with HEPES buffer via buffer replacement with a pipette, ensuring that the sample was constantly hydrated. Samples were then transferred to a MultiMode 8 AFM instrument equipped with a Bruker Nanoscope V controller for topographic imaging. Fluid imaging was run in contact mode at the lowest force set point using thermally stabilized cantilevers using silicon nitride AFM cantilevers (MLCT-BIO-DC, cantilever C, Bruker Probes, Camarillo, CA) with a nominal spring constant of 0.01 N m^–1^ (Bruker AFM probes, Camarillo, CA) within a fluid cell filled with HEPES buffer.^12,42^ More commonplace oscillatory modes fail due to the low modulus of the microgels’ surface coupling with the probe oscillations. Images were acquired at 512–640-pixel resolution and processed using Bruker NanoScope Analysis v3.0.

### Apparent Viscosity of Microgel Dispersions

A modular compact rheometer (MCR-302, Anton Paar, Austria) was used to measure the viscosity of PoPS and PoPM dispersions (the latter at 50% microgel content) at 25 °C. Cone-and-plate geometry (CP50-2, diameter: 50 mm, cone angle: 2°) was used for measurements of PoPM dispersions, while a concentric cylindrical geometry (inner diameter of cup: 24.5 mm, diameter of bob: 23 mm) was utilized for PoPS measurements. Viscosities were measured at shear rates from 1 to 1000 s^–1^, and a minimum of three replicates were measured for each sample.

### Interfacial Tension

Interfacial tension was measured using an OCA 25 (Dataphysics Instruments, Germany) drop shape tensiometer. PoPM dispersions were diluted to 0.01 wt % protein concentration, and a pendant drop of 22 μL volume was formed at the tip of a syringe (DS500 GT, 1.65 nm) tetradecane within a glass cuvette at 22 °C. Each pendant drop was monitored for 1800 s, and the interfacial tension over time was calculated via the Young–Laplace equation fitted to the extracted droplet shape using dpiMAX software. The density of microgel and protein dispersions was assumed to be equivalent to water (0.9982 g cm^–3^), while the density of tetradecane was 0.7628 g cm^–3^. Measurements were conducted in triplicate and duplicated (i.e., *n* = 3 × 2).

### Interfacial Shear Rheology

Interfacial small-amplitude oscillatory shear rheology was measured using a modular compact rheometer (MCR-302, Anton Paar, Austria) fitted with bicone geometry (BiC68-5) at 25 °C. Raw viscoelastic data were numerically analyzed to consider the influence of the upper and lower phases to correctly calculate values of the interfacial shear moduli (*G*_*i*_′ and *G*_*i*_″).^43^ The lower fluid aqueous phase was PoPS or PoPM dispersions diluted to 0.01 wt % protein; tetradecane was then gently added to form the upper fluid oil layer. At this dilution, as for the interfacial tension measurements, it is safe to assume that the density and bulk viscosity of the aqueous phase are equal to that of pure water. Samples were monitored over 15 h (54,000 s) at fixed angular frequency and strain of 6.283 rad s^–1^ and 1%, respectively, after which amplitude sweeps were measured at 0.01–100% strain, maintaining a constant angular frequency of 6.283 rad s^–1^. Each measurement was duplicated and conducted on at least two separate samples (i.e., *n* = 2 × 2).

For interfacial rheological studies where the temperature was varied, after the initial 15 h period, samples were heated to 40 or 70 °C at a rate of 0.08 °C s^–1^, held at these temperatures for 10 min, and then cooled back to 25 °C and monitored to see if the preheated values of interfacial moduli were recovered. Measurements were duplicated for each sample type and temperature.

### Circular Dichroism (CD)

Circular Dichroism was utilized to investigate the secondary structure of both the PoPS and PoPM, diluted to 0.02 wt % protein. A Chirascan Plus (Applied PhotoPhysics Spectropolarimeter, Leatherhead, U.K.) instrument was used, generating far-UV spectra between 180 and 260 nm at a 2 nm bandwidth and 1 nm step size. Measurements were made in 1 mm path length quartz cuvettes at 20 °C. In the Results section, no data are shown below 200 nm because, in this region, there was much noise due to adsorption by the HEPES buffer.

### Statistics

Means and standard deviations are reported from at least three readings on triplicate measurements. One-way analysis of variance (ANOVA) (Duncan test) was conducted using SPSS statistical software (version 28) to identify the significant differences between the tested samples. A difference was defined as significant when *p* < 0.05.

## Results and Discussion

### Characteristics of Parent PoP Gels

**Figure 1:**
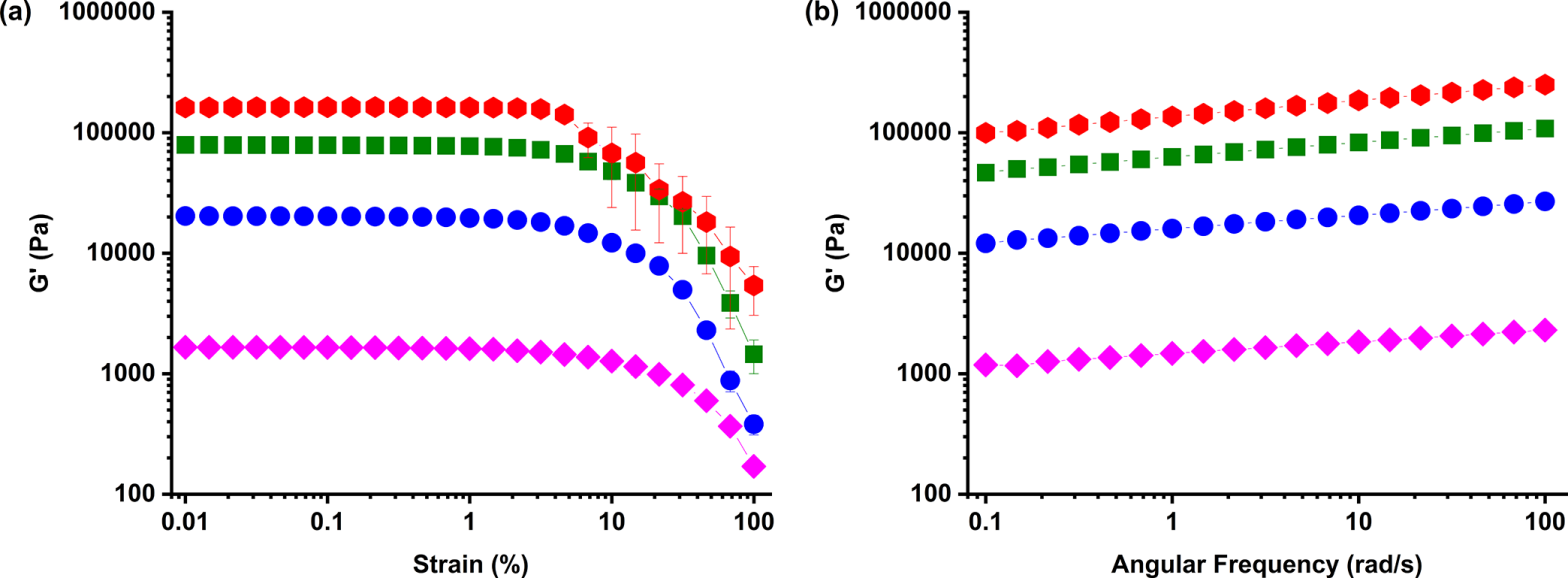
Strain sweeps (a) at a constant angular frequency of 6.283 rad s^–1^ and (b) frequency sweeps at a constant strain of 0.1%; *G*′ (solid symbols) are shown for potato protein “parent” gels at 5 wt % (pink diamonds), 10 wt % (blue circles), 15 wt % (green squares), and 18 wt % (red hexagons) concentration.

**Figure 2:**
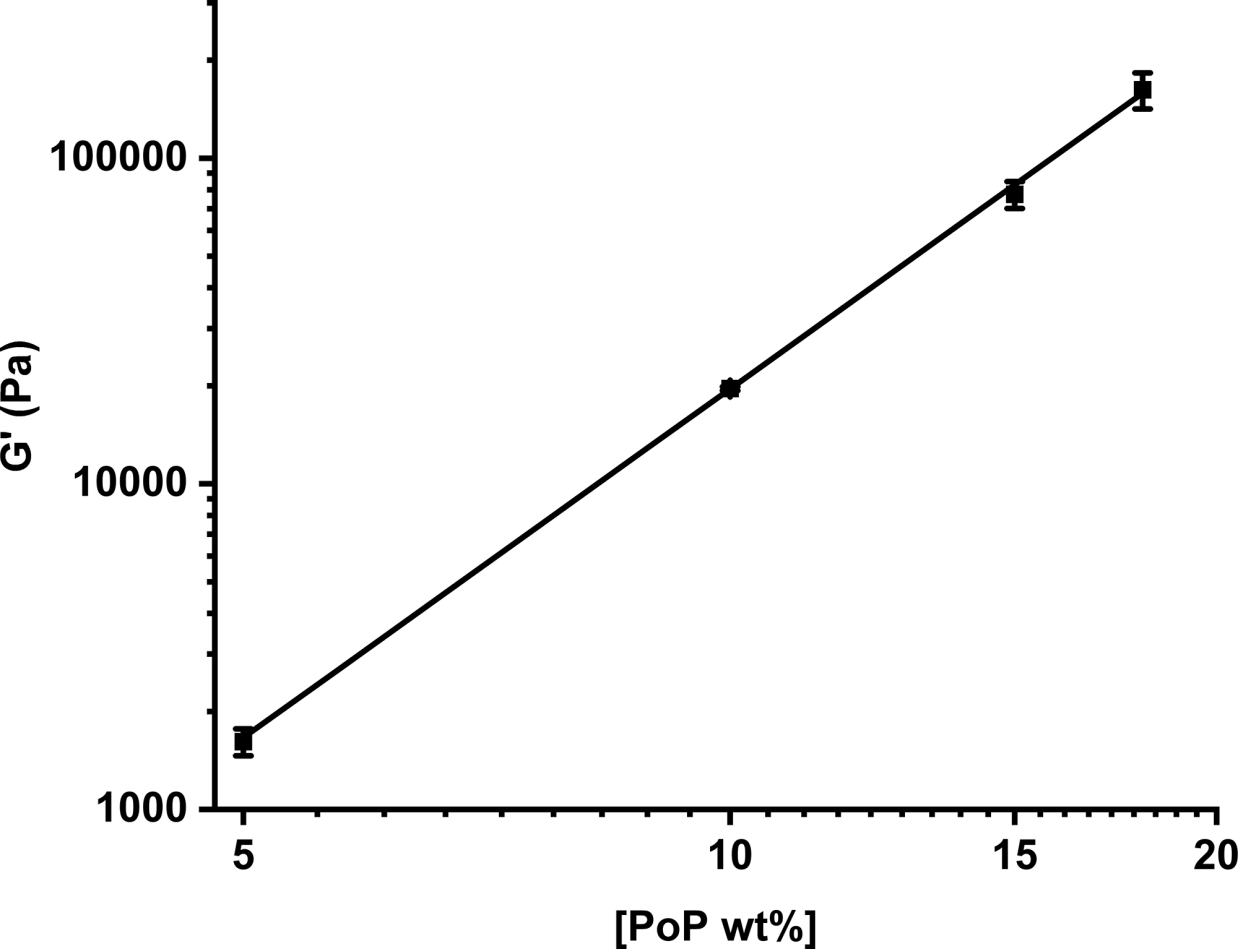
Bulk moduli (*G*′) of parent gels taken at 1% strain plotted against their potato protein concentration ([PoP]). The slope indicates a power law exponent (*t*) of 3.56.

As yet, it is impossible to unambiguously characterize the bulk rheology of submicron-sized microgel particles; however, it was assumed that the rheological measurements on the parent gels formed in situ in the rheometer should reflect to some extent the mechanical properties of the PoPM. Figure 1a displays a strain sweep conducted on these parent bulk gels and clearly shows a significant difference in storage moduli (*G*′) when the PoPS concentration [PoPS] increases. Within both Figure 1a,b, increases in storage moduli of an order of magnitude can be seen when [PoPS] increases from 5 to 10 wt %. Similarly, large increases are also observed from 10 to 15 wt % and between 15 and 18 wt %, with values reaching >10^5^ Pa. Analogous increases in loss moduli (*G*″) are shown in Figure S1a,b. Figure 2 demonstrates the increase in bulk modulus of the parent gels with concentration, which follows a power law dependence with an exponent *t* of 3.56 ± 0.06, a value that describes the origin of elasticity within the material. Values of *t* ≤ 1.5 imply entropic elasticity (rubber elasticity theory) governed by the random conformation and freedom of motion of molecular scale chains, typical of synthetic polymer gels. Values around 2 describe the enthalpic bending of cross-linked structures such as fibers or linear assemblies, where the rigidity or persistence length of the structure governs elasticity. A value of *t* ≫ 2 is often seen in biopolymer gels, thought to be due to the high stiffness of the primary structure of the gel. In this case, globular proteins retain much of their structure but are bonded through partial denaturation and exposure of hydrophobic residues, forming local rigid assemblies, which create a network of semiflexible rods. Another reason is the cross-links themselves, the junction zones within the gel, are no longer single-point molecular contacts but large stiff assemblies with multiple connections. Values of *t* for biopolymer gels are typically 2.4–4.2, meaning that the potato protein gel has relatively rigid links between network junction points.^44,45^

Across all samples, the linear viscoelastic regime (LVER) remained similar, with *G*′ starting to decrease beyond ca. 5% strain. However, the gels at [PoPS] = 18 wt % showed a steeper fall in *G*′ beyond the LVER, indicative of a more brittle gel.^46^ Frequency sweeps on the gels (Figures 1b and S1b) show that both *G*′ and *G*″ were largely independent of frequency, confirming their largely solid-like characteristics.^47^

These PoP bulk gels have elastic moduli similar to those measured elsewhere for PoP at pH 7.0.^18,26,29^ Compared to whey protein gels, cross-linking in PoP gels is thought to differ due to its dependence on hydrophobic linkages.^26^ The structure of patatin contains only one free thiol group and no internal disulfide bridges, which restricts the molecule from forming a covalently bonded gel network via disulfide bond rearrangement.^34,48^ This may explain the generally lower fracture strain for PoP gels. Hydrophobic bonds are more sensitive to heating, and so patatin undergoes a greater extent of unfolding, also explaining PoP’s relatively low denaturation temperature and, therefore, the ability to gel at lower temperatures as well as lower bulk protein concentrations.^23,24,29^ All of this also means that PoP has a higher solubility compared to many other plant proteins.^25^

### Characteristics of Microgel Dispersions

**Figure 3:**
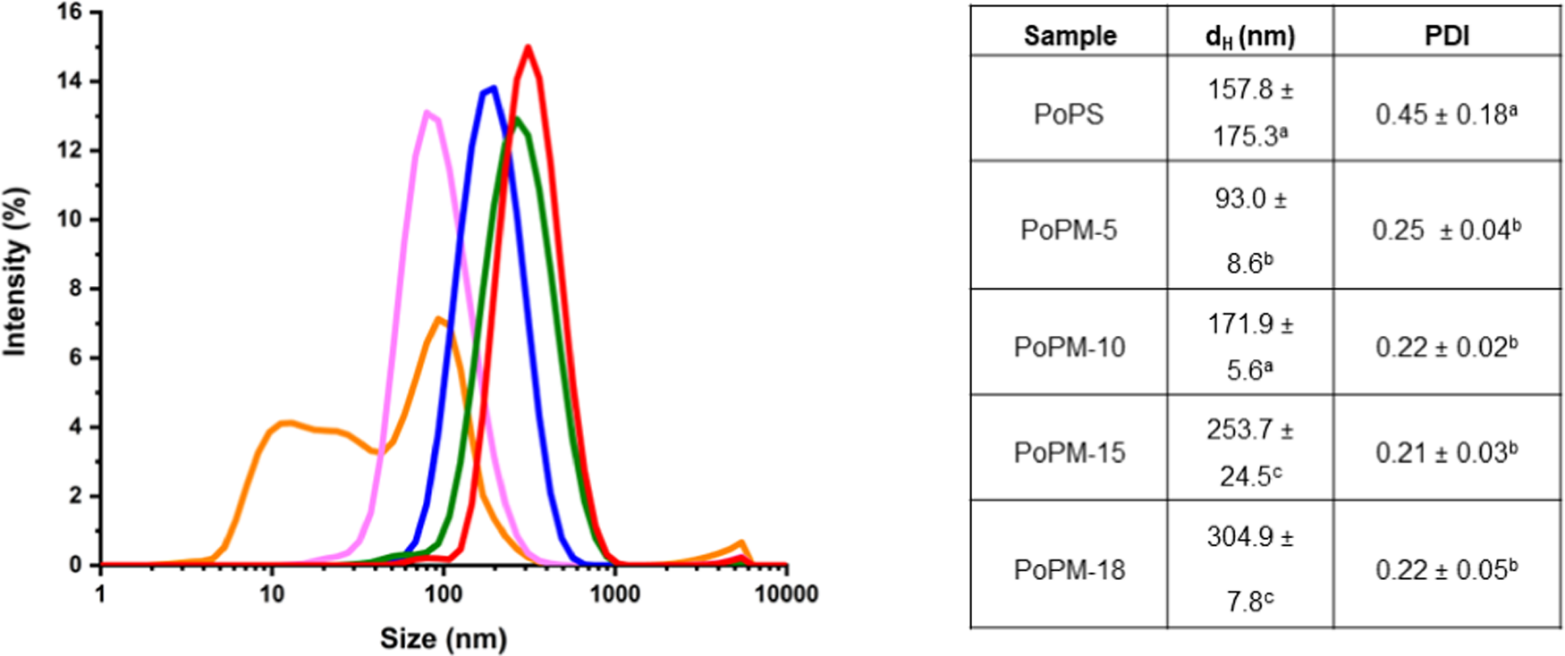
Mean size distributions of PoPM-5 (pink), PoPM-10 (blue), PoPM-15 (green), and PoPM-18 (red) compared to PoPS (orange). The inset (table) shows the corresponding mean hydrodynamic diameter (*d*_H_) and polydispersity (PDI). Different superscript letters (a–c) indicate significant (*p* < 0.05) differences between olydispers and PDI values.

Figure 3 displays the narrow size distribution of the PoPM samples, with a peak at ca. 70 nm for PoPM-5, with the peak of the distributions increasing with increasing [PoPS] up to ca. 400 nm for PoPM-18. At higher [PoPS], the density of cross-links within the PoPM is expected to be higher,^24^ and the mechanical strength of the particles, like that of the parent gels, is higher, which in turn makes them more difficult to break up into smaller particles. This is reflected in the roughly proportional relationship between wt% (and hence overall density and cross-link density) with microgel diameter.

Compared to PoPM, PoPS itself shows significantly higher polydispersity, with peaks around 10 and 100 nm. The 10 nm peak is likely to be the individual patatin monomers (nonspherical, with a radius of 2.5 nm and length of 9.8 nm^49^), and the 100 nm peak indicating significant protein aggregates are present, as observed elsewhere.^18,36^ This may also explain the statistically similar averages of hydrodynamic diameters for PoPS and PoPM-10, as aggregates of PoPS could reach diameters equivalent to those of PoPM-10.

**Figure 4:**
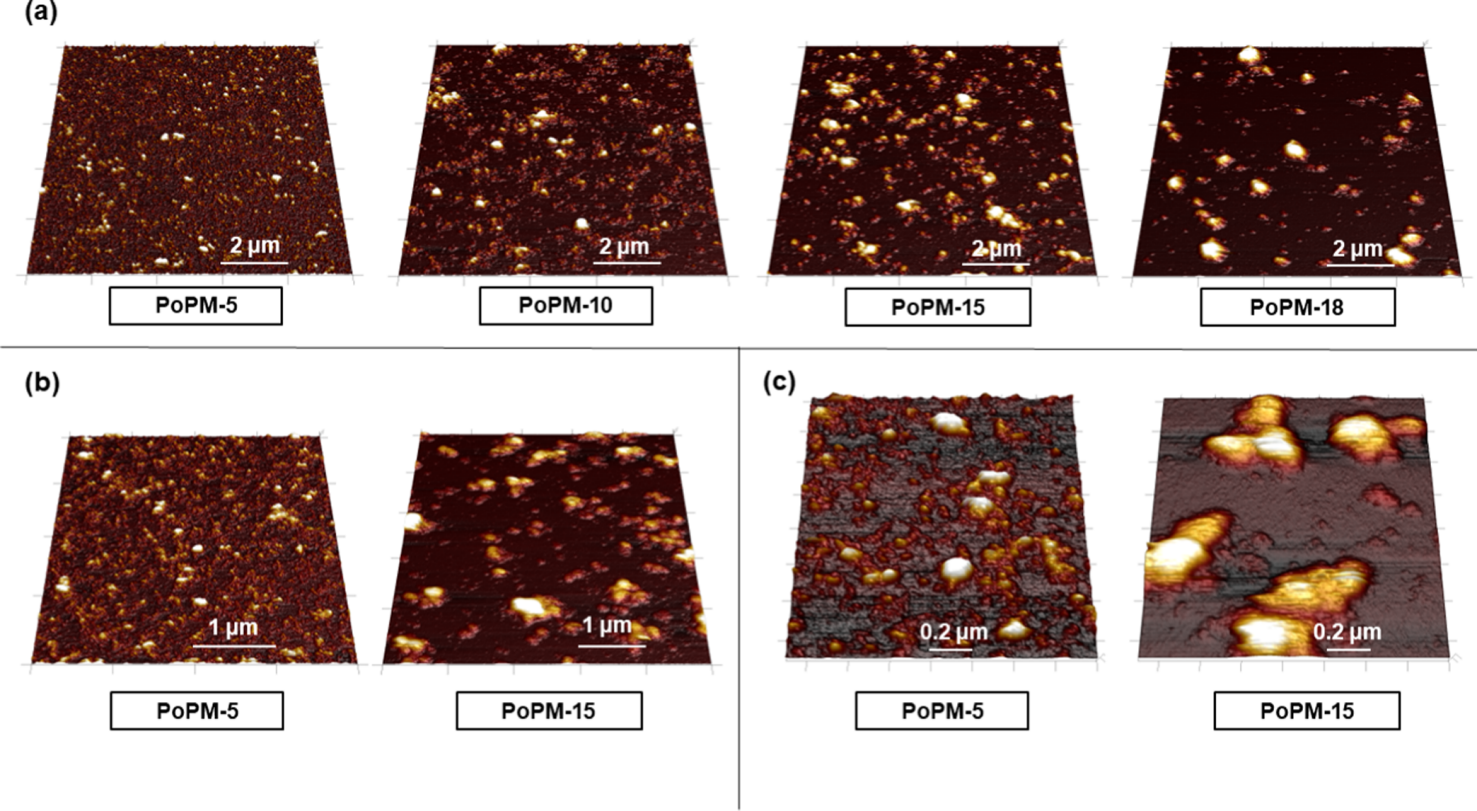
AFM images of microgels at pH 7.0 adsorbed onto silicon: (a) PoPM-5, PoPM-10, PoPM-15, and PoPM-18 at scan areas of 10 μm × 10 μm; (b) images of PoPM-5 and PoPM-15 at scan sizes of 4 μm × 4 μm and 5 μm × 5 μm, respectively, and (c) images of PoPM-5 and PoPM-15 over areas of 1.5 μm × 1.5 μm.

Imaging of the PoPM via AFM demonstrates their morphology and aggregation, as shown in Figure 4. A range of particle sizes is evident but, overall, complementary to the distributions obtained via DLS (Figure 3). The PoPM appear to have a near-spherical shape but with some rough edges that might be expected as a result of the vigorous “top-down” shearing production method.^18^ (In contrast, the PoPS aggregates have a more smooth spherical shape but of much smaller size; see Figure S2.) It should be noted that both the DLS particle size distributions and AFM images were obtained at high dilution, whereas PoP is known to readily aggregate at higher [PoPS].^28^ The AFM images (at the same overall [PoPS] = 0.01 wt %) show that the higher the protein content of the PoPM, the more aggregated they are. For example, PoPM-15 and PoPM-18 show aggregates with diameters ≈1 μm.

To assess the secondary structure of PoPS samples compared to those of PoPM, CD spectra were obtained in the far UV, as shown in Figure S3. This provided further confirmation of denaturation, as the proportion of α-helices, which can be seen as negative peaks at approximately 210 and 220 nm for PoPS, tend to disappear for the PoPM.^50,51^ These are replaced by a singular peak, indicative of β sheet structure,^36^ which is increasingly defined in the range of 215–220 nm for the PoPM formed at higher [PoPS]. In fact, the weakest microgel sample (PoPM-5) actually yielded an absorbance at this wavelength that was even lower than that for PoPS, possibly indicating an alternate aggregated state of PoP as a result of heating at this low bulk concentration.

**Figure 5:**
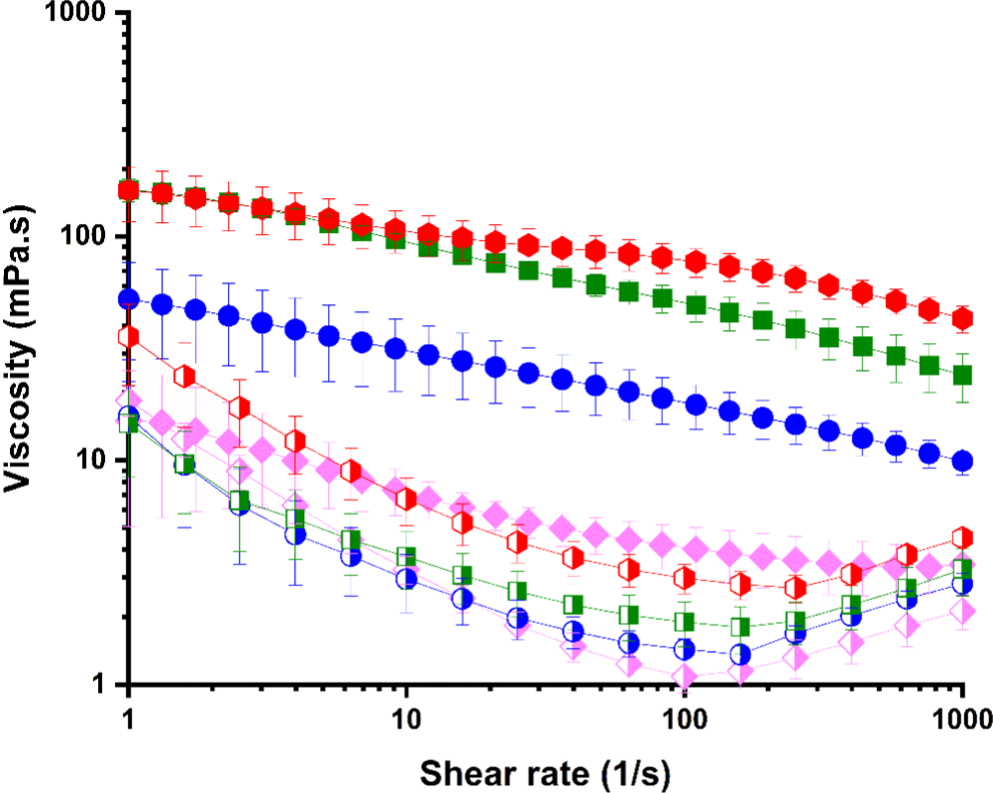
Bulk viscosity of PoPM dispersions at 50 vol % (filled symbols): PoPM-5 (pink diamonds), PoPM-10 (blue circles), PoPM-15 (green squares), and PoPM-18 (red hexagons); compared to that of PoPS (half-filled symbols) at equivalent overall concentrations of PoP, i.e., 2.5 wt % (pink diamonds), 5 wt % (blue circles), 7.5 wt % (green squares), and 9 wt % (red hexagons).

The rheological properties of the PoPM (at a particle concentration of 50 vol %) were studied and compared to that of PoPS at the same overall [PoPS], as shown in Figure 5. It is seen that at PoPM-10, -15 and -18 had significantly higher viscosities at all shear rates compared to the corresponding PoPS. Both solutions and microgels were significantly shear thinning, the solutions more so up to a shear rate of around 100 s^–1^, as observed elsewhere.^27^ This shear thinning indicates breakup of structure—networks and aggregates of both PoPM and PoP molecules, held together via weak bonds.^40,52^ The apparent slight increase in viscosity of PoPS at shear rates >100 s^–1^ is most likely an artifact of the instrument at these low stresses and high shear rates. The trend in viscosity versus shear rate of PoPM-5 is closer to that of the PoPS samples, probably because the PoP-5 parent gel was near the sol–gel transition, and so the PoPM-5 dispersion was closer to a dispersion of large protein aggregates, like the PoPS. Therefore, it is likely that a critical protein concentration beyond 5 wt % is required for “true” gel formation.

The rheological data thus highlight the capacity of the PoPM to modify the viscosity by an order of magnitude at just 50 vol %, the range also depending upon the type of PoPM (i.e., [PoPS] used to make the PoPM). No doubt, the range would be considerably widened by varying the vol % of microgels in the system, as shown elsewhere.^12,40^ The capacity of microgels to interpenetrate has been suggested to allow for more reversible shear thinning, i.e., shear is not thought to completely destroy the individual microgel particles.^19,52^ For PoP solutions (PoPS), which perhaps ought to be more correctly described as dispersions of PoP aggregates, the interparticle interactions are less likely to be regained post shearing (though we have not tested this yet).

### Characterization of Microgels at an O–W Interface

**Figure 6:**
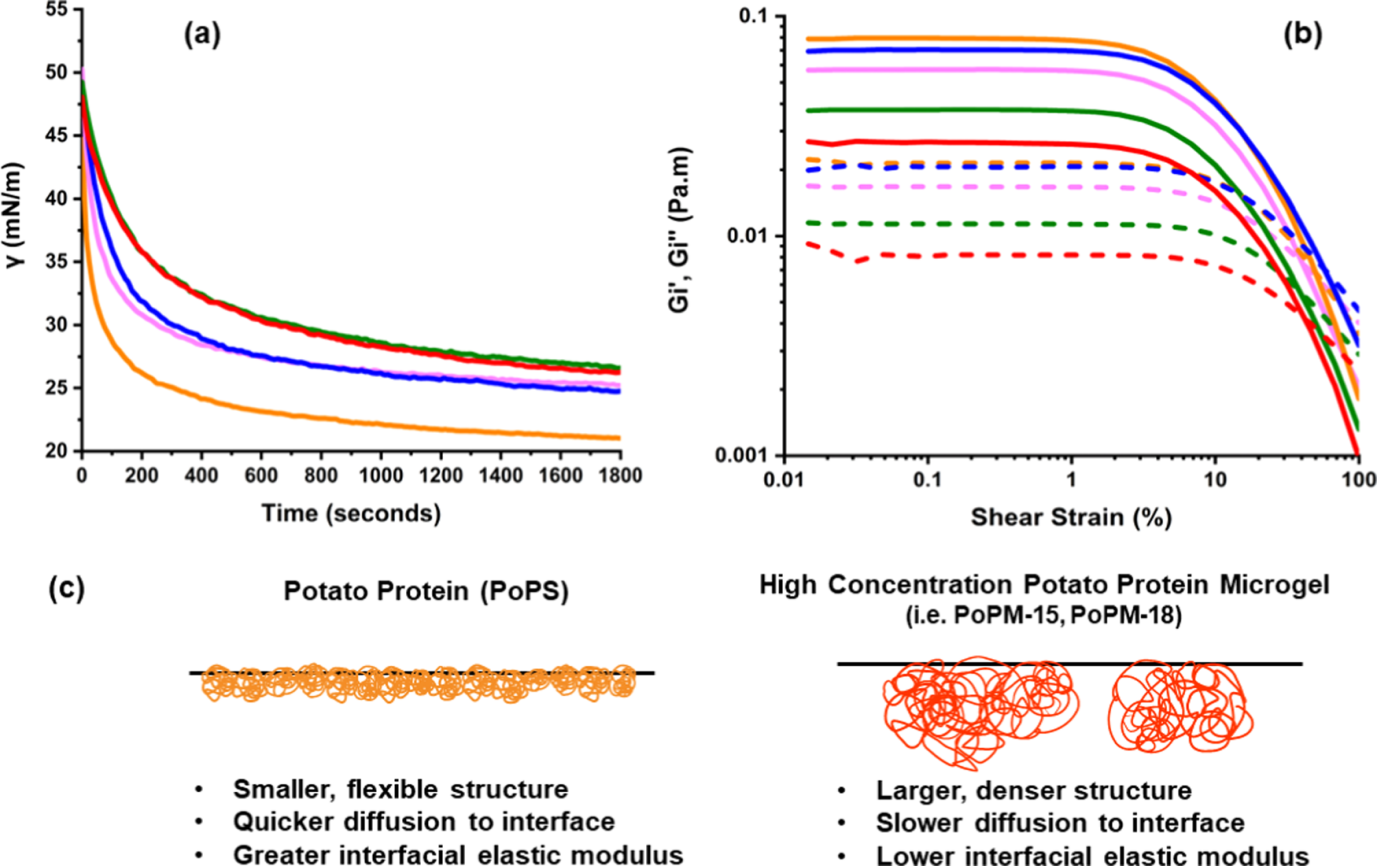
(a) Interfacial tension (γ) at the tetradecane–water interface, measured against time. (b) Interfacial storage moduli (*G_i_*′) (solid lines) and interfacial loss moduli (*G_i_*″) (dotted lines) against strain at the oil–water interface after 15 h of equilibration time, measurements conducted at a constant frequency of 1 Hz. Both panels (a) and (b) shown for PoPM samples at 5 wt % (pink), 10 wt % (blue),15 wt % (green), and 18 wt % (red) concentration compared to PoPS (orange). (c) Illustration demonstrating the potential structure of interfacial packing for potato protein (left) compared to potato protein microgels made from high protein concentration parent gels (right).

As shown in Figure 6a, all PoPM dispersions and PoPS (diluted to equal overall [PoPS] = 0.01) displayed a sharp decrease in interfacial tension (γ) within the first 400 s, demonstrating high affinity adsorption to the O–W interface. Following this sharp drop in γ, the values of γ continued to decrease more slowly and almost leveled out after 1800 s (30 min). All PoPM showed higher values of γ than the PoPS throughout, by at least 5 mN m^–1^, which is most likely simply a kinetic effect due to the larger size of the PoPM and their aggregates and, therefore, their slower diffusion to the interface (see Figure 6a and Table S1), as has been reported elsewhere.^15,16^

Figure 6a shows that microgels of lower PoP content (PoPM-5 and PoPM-10) appeared to be quicker to adsorb and reduce γ than those of higher PoP content (PoPM-15, PoPM-18). These differences may be due to several factors. First, the PoPM-15 and PoPM-18 samples of higher PoP concentration are somewhat larger than the PoPM-5 and PoPM-10 (see Figures 3 and 4), which will slow down their diffusion to the interface. The influence of size is further illustrated in Table S1, which shows estimated diffusion coefficients^53^ compared with the gradient of initial decrease in γ (over the first 400 s studied). It is clear that the diffusion constant decreases with increasing size, which is also consistent with the lower values for the rate of γ decline for the larger microgels. Additionally, assuming that the cross-link density of the PoPM-15 and PoPM-18 reflects that of their parent gels, these microgels are expected to be stiffer and, therefore, slower to unfold and adhere to the interface if some unfolding is necessary for exposure of more hydrophobic groups at their surface to induce adsorption.^16,54^

At the same time, it is important to consider the role of free, ungelled proteins within the microgel dispersions.^55−57^ Not all PoP originally present may be incorporated into the parent gels, and furthermore, there may be some release of individual protein molecules and/or relatively small aggregates of them on the mechanical disruption of the gels to PoPM. These lower *M*_w_ species will therefore be present in the PoPM dispersions to compete with them for adsorption and adsorb faster, as pointed out above. AFM imaging, as shown above in Figures 4 and S2, suggests that all PoPM samples led to the development of a protein film formed of particles smaller than microgel size, covering the substrate.

Once at the interface, proteins unfold to adopt lower free energy conformations, revealing otherwise hidden amino acid residues that may then more readily form intermolecular cross-links via hydrophobic interactions, H-bonding, salt bridges, and even sulfhydryl groups that can form new disulfide bonds via sulfhydryl-disulfide exchange reactions.^4,5,58^ It is not always clear if one type of intermolecular bonding dominates between proteins or protein-based microgels at the interface,^59^ but these intramolecular interactions generally facilitate the development of a stiff viscoelastic network at the surface of droplets, bubbles, etc., that is associated with higher colloidal stability.^2,60^ Interfacial shear rheology measurements are highly sensitive to the development of these interfacial networks,^54^ and so this was also measured.

For adsorbed protein molecules, the interfacial shear rheology can continue to evolve over very long time scales as the molecules continue to change their conformation and bonding. Due to their larger size and already highly cross-linked nature, this may take place over an even longer time frame for microgels.^21^ Here, we measured interfacial moduli after 15 h adsorption; their development over this time period is displayed in Figure S4. Interfacial amplitude sweeps, shown in Figure 6b, were conducted on PoPS and PoPM at a constant frequency of 1 Hz. All samples produced similar LVER regions: a strain of ca. >2% led to gradual shear thinning, typically described as type 1 behavior.^61^ This prolonged region of yielding, see Figure 6b, led to eventual crossover in *G*_*i*_″ and *G*_*i*_′ beyond 10% strain, i.e., exhibiting “strain softening,”^61^ which has been suggested to reflect the rearrangement and interaction of proteins at the interface.^60,62^

A recent study evaluating patatin-rich and protease inhibitor-rich potato protein samples reported a weak strain overshoot (exhibited as an increase in *G*_*i*_″, indicating that the rate of bond formation is slightly higher than bond breakage^61^) at the yielding strain of ca. 2–3% strain in both samples.^31^ This may be correlated to more interconnected interfacial structures^63^ and implies that systems rich in specific potato protein subunits^31^ may yield comparatively more brittle interfacial layers than observed with our PoPM or PoPS. However, the bulk protein concentration used in this study was higher, which will also alter *G*_*i*_′ and *G*_*i*_″. Interestingly, patatin-rich samples were suggested to be more suitable to promote dynamic emulsion stability.^31^ The influence of gelation on potato protein interfacial behavior has not yet been considered in the literature; thus, our current study is crucial to ensure that potato protein functionality can be maximized.

Within commercial potato protein isolates such as that used here, there is a complex mixture of protein fractions,^39^ which may all interact at the interface. As previously discussed, potato protein has high surface hydrophobicity,^29^ which suggests that due to intermolecular interactions between the proteins, they would adsorb at the interface already in a somewhat aggregated state.^5,28^ It can be seen in Figure 6b that PoPS leads to higher interfacial moduli than any of the PoPM. This is somewhat surprising, given results with other globular proteins converted into microgels are the other way round,^64^ it implies that the ungelled potato protein exhibits stronger and/or more prevalent attractive lateral interactions when adsorbed at the interface than the PoPM. Possibly, hydrophobic patches on the individual PoP molecules, or their aggregates, enable the formation of a “granular two-dimensional” close-packed solid layer at the interface.^60^ This is illustrated schematically in Figure 6c. Intermolecular disulfide bond cross-linking is often associated with high values of interfacial shear viscoelasticity,^4^ but this is not a prerequisite for high moduli; multiple H-bonds, plus strong adsorption and unfolding that lead to interfacial jamming also lead to strong films and promote colloidal stability.^54,60^

Figure 6b also shows that the interfacial moduli of the adsorbed PoPM were lower when the [PoPS] of the parent gels is higher, which is in contrast to the higher elastic moduli seen for these parent gels in Figure 1. This trend in *G*_*i*_″ and *G*_*i*_′ might be attributed to the larger sizes of microgels originating from parent gels of higher [PoPS], see Figure 3, but may also be associated with their flexibility at the interface. It has been shown that interfacial elasticity tends to be higher for adsorbed films of smaller microgels,^65^ which was linked to increased packing and mobility of microgels in the interface. This dependence on size has also been reported in interfacial systems of protein monomers versus larger aggregates of the same protein.^66^ At the same time, as mentioned above in connection with the trends in γ (Figure 6a), it is likely that there is competition at the interface between PoPM, PoPS, and PoPM fragments in between the sizes of the latter.

For whey protein-derived microgels (WPM), greater interfacial dilatational elasticity has been reported when compared to the native protein,^55^ while the high stability of WPM-stabilized emulsions has been attributed to a tendency toward bridging flocculation as opposed to coalescence.^56^ Lysozyme-based microgels have been found to rapidly aggregate at the interface, leading to a mixture of clustered, rigid patches and empty regions.^67^ This surface heterogeneity of adsorbed microgel systems is obviously dependent on protein type and is similar to observations of individual globular proteins, which have shown eventual displacement of protein clusters from the interface.^58^

For microgels of plant protein origin, pea protein microgels (PPM) have also been found to display aggregation at the interface,^17^ but it has been proposed that they are less deformed at the interface than WPM.^37^ For microgels fabricated from soy protein, lower values of dilatational elasticity have been observed cf. the native protein, which was also attributed to the greater rigidity of microgel particles.^16^ On the other hand, it has been proposed that a balance between wettability, unfolding, and compactness of structure gives the optimum packing of particles at the interface,^16^ which in turn should translate to optimum emulsion stability. For the PoPM studied here, it appears that when they are formed from stronger gels (of higher [PoPS]), the particles may be so rigid that this inhibits their flexibility at the interface and limits in-plane interactions, leading to lower *G*_*i*_″ and *G*_*i*_′ (see Figure 6c). Thus, the PoPM behave more like “inert” particles and create a less interconnected, weaker interfacial structure.

This agrees with the view that an optimal level of cross-linking is probably required to produce effective levels of film flexibility and mechanical strength.^68^ If particles are overly cross-linked, there may be the risk of worsening the interfacial flexibility of the plant protein^2^ and creating even further aggregated and brittle structures. Thermodynamically, more stable protein structures have been shown to adsorb more slowly and produce films with lower viscoelasticity.^62^ Thus, the combination of size and deformability appears to be critical in the formation of strong, viscoelastic interfacial layers to promote emulsion stability.

**Figure 7:**
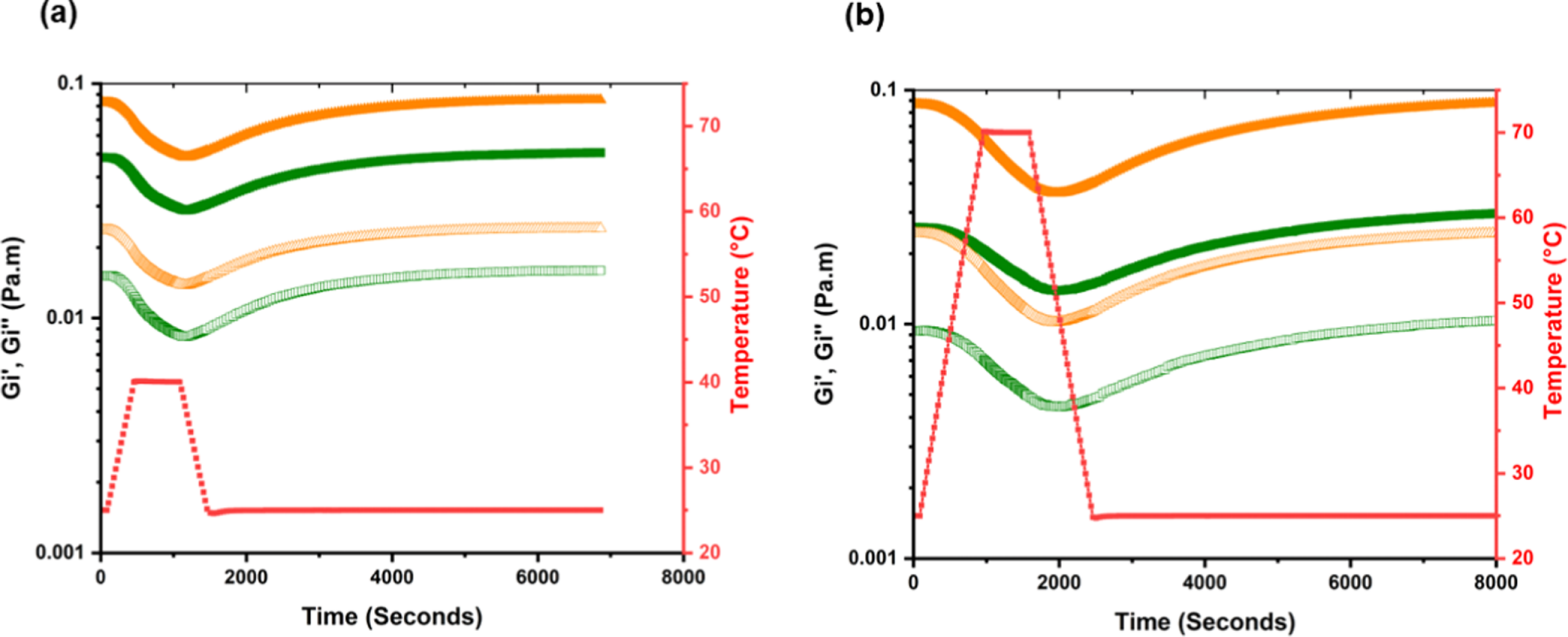
Oscillatory interfacial shear rheology versus temperature sweeps for (a) increasing from 25 to 40 °C and back again (b) increasing from 25 to 70 °C and back again for PoPM-15 microgels (green squares) were compared to PoPS (orange triangles). Interfacial storage moduli (*G*_*i*_′) (solid shapes) and interfacial loss moduli (*G*_*i*_″) (empty shapes) measured at the oil–water interface after 15 h equilibration time; temperature change is displayed with red squares.

One way of varying microgel flexibility is via temperature, which is also important to test because heat stability is a common challenge for many colloidal systems, which might be due to protein denaturation and aggregation on heating, whereas protein microgels may be less affected due to their being formed from predenatured protein.^69^Figure 7 shows the effect of heating adsorbed PoPM-15 and PoP at the O–W interface: heating from room temperature to 40 or 70 °C. These temperatures were chosen to explore the influence of bond breakage that may occur in processing environments, any changes on heating to 40 °C being expected to be reversible, whereas heating to 70 °C might induce further irreversible changes for both PoP and PoPM.^70^Figure 7 demonstrates that although the moduli of the 15 h old films decreased when the system was subjected to 40 and 70 °C, the values were recovered after cooling for both PoP and PoPM samples. Figure 7a shows that the initial *G*_*i*_′ values (0.083 and 0.048 Pa m for PoP and PoPM, respectively) were regained 90 min after heating to 40 °C (to values of 0.085 for PoP and 0.051 for PoPM). Figure 7b shows that post heating to 70 °C, *G*_*i*_′ values (of 0.087 for PoP and 0.026 for PoPM) were also recovered within 90 min (to values of 0.088 and 0.03 for PoP and PoPM, respectively). For both temperatures, samples exhibited a slight increase post heating, thus further rearrangements may potentially occur with time, which could promote the development of even higher moduli over longer time scales.^21^ However, since most heat-set protein gels are not thermo-reversible, the risk of syneresis should be considered if higher temperatures/longer heating times were applied,^33^ leading to loss of water from the microgels. Surprisingly, there have been very few other studies of the effects of heating on adsorbed or nonadsorbed protein-based microgels despite its technological significance. The similar response for both PoPM and PoP suggests that the bonding affected by these temperature increases is the same in both cases. Small increases (e.g., up to 40 °C) in temperature are likely to only promote hydrogen bond breakage.^33^ PoP interactions have been reported to be dominated by hydrophobic bonding, with a small contribution from disulfide bonds at pH 7.^24,48^ In Figure 7b, the sharp drop in moduli seen at 70 °C implies that stronger and/or more numerous bonds are broken than those at 40 °C, where the decrease is less distinct (Figure 7a). The fact that the moduli are largely recovered on cooling means that this bonding is reversible and, therefore, most likely hydrophobic bonding and H-bonding. Thus, potentially, the mechanism of aggregation between the PoPM and PoP molecules at the interface is the same. However, the starting and final values of interfacial moduli *G*_*i*_′ and *G*_*i*_″ are still different because the compact structure of the PoPM limits the extent of their intermolecular interactions, as discussed above.

## Conclusions

This study describes an investigation into the role of protein concentration on potato protein microgel interactions in the bulk and when they are adsorbed at an O–W interface. It is clear that increasing potato protein concentration within the microgels (produced from parent macrogels of increasing strength) has a significant influence on microgel behavior. Within bulk solution, microgels produced from macrogels of 10 wt % concentration and greater exhibited high capacity for viscosity modification, compared to ungelled potato protein solutions.

Although the bulk elastic modulus of parent gels increased with the potato protein concentration, the opposite trend was seen for interfacial elasticity microgels. Surprisingly, it was observed that once these parent gels were sheared, the particles produced from gels of the highest concentrations yielded the lowest interfacial elastic moduli. These stronger, larger microgels likely lack flexibility and diffuse to the interface at a slower rate, whereas, upon adsorption, they appear to be less capable of forming lateral interactions and may instead yield weaker, less interconnected interfacial films.

To the authors’ knowledge, this is the first time that interfacial monolayers of potato protein microgels have been studied. Microgel interfacial film strength can be decreased on heating, but this decrease is completely reversible on cooling. The same behavior was demonstrated for nonmicrogelled potato protein, which implies that the same type of protein–protein bonding is present at the interface. Thus, the surface hydrophobicity of potato protein may dictate its lateral interactions in both ungelled and microgel forms, while heating offers one way of tuning this interfacial behavior.

Future studies should therefore aim to further evaluate the mechanical strength (e.g., modulus) of biopolymeric microgels (in particular those created from components of plant origin) and their behavior under varying conditions, for example, within alternative pH environments or at air–water interfaces. Investigations of biopolymeric microgel monolayers utilizing dilatational rheology and Langmuir–Blodgett depositions would also aid in the optimization of emulsion stabilizers and will form part of our future studies. Additionally, the role of protein type and heterogeneity within samples should be explored in greater depth to clarify the parameters controlling the formation of viscoelastic monolayers to confirm whether these findings for potato protein microgels would also be apparent for microgels produced from different plant protein sources. These findings will be key in the development of sustainable stabilizers in a range of colloidal applications.

## Supporting Information Available

Frequency and amplitude sweeps of the bulk “parent” gels displaying both storage and loss moduli (Figure S1); atomic force microscopy images of ungelled potato protein (Figure S2); far-UV spectra for ungelled potato protein compared to microgel (Figure S3); interfacial shear moduli over time (during a 15 h equilibration period) for ungelled potato protein compared to microgel samples at a tetradecane–buffer interface (Figure S4); and a table displaying estimated diffusion coefficients for samples based on their interfacial tension and particle size (Table S1) (PDF)
