# Supplementary material for: Bulk and Interfacial Behavior of Potato Protein-Based Microgels
Source: Langmuir. 2024 Oct 1;40(41):21341–51. doi: 10.1021/acs.langmuir.4c01785 (PMC11483775; doi:10.1021/acs.langmuir.4c01785)
Supplement: Supplementary file 1 — la4c01785_si_001.pdf [file la4c01785_si_001.pdf]

Supporting Information

Bulk and interfacial behaviour of potato protein-based microgels

Daisy Z. Akgonullu[1], Nicholas O'Hagan[1], Brent S. Murray[1], Simon D. Connell[2], Yuan Fang[3], Bruce Linter[4], Anwesha Sarkar[1]*

[1] Food Colloids and Bioprocessing Group, School of Food Science and Nutrition, University of Leeds, UK

[2] Molecular and Nanoscale Physics Group, School of Physics and Astronomy, University of Leeds, UK

[3] PepsiCo, Valhalla, New York, NY, USA

[4] PepsiCo International Ltd, Leicester, UK

*E-mail: A.Sarkar@leeds.ac.uk

Number of pages: 5

Number of figures: 4

Number of schemes: 0

Number of tables: 1

Contents

Figure S1. Oscillatory rheology sweeps **(a)**, strain amplitude sweeps at constant angular frequency of 6.283 rads^{-1} and **(b)** frequency sweep at constant strain.................................**S1**

Figure S2. Atomic force microscopy images of potato protein illustrating particles in 3-D over a scan area of **(a)** 10 microns **(b)** 2 microns and **(c)** 0.5 micron..**S2**

Figure S3. Far UV Spectra...**S3**

Figure S4. Interfacial shear moduli (G_i' and G_i'') against time at the oil-water interface for 15 h equilibration time, measurements conducted at a constant frequency of 1 Hz. **S4**

Table S1. Table displaying the initial decrease in interfacial tension in comparison to diffusion coefficients (D) and particle size **S5**

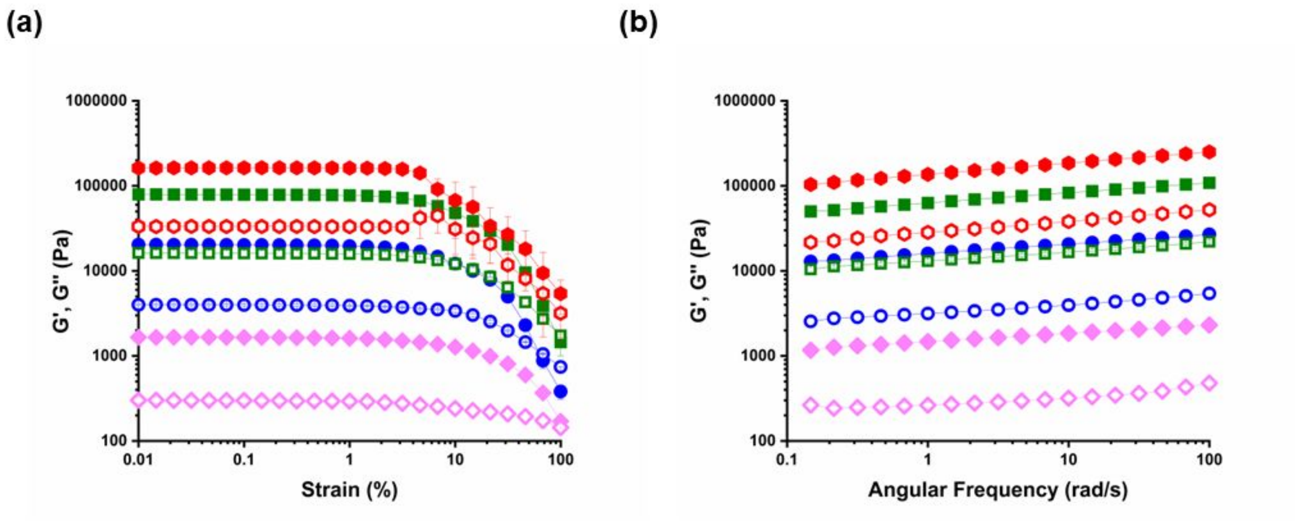

Figure S2. Oscillatory rheology sweeps **(a)**, strain amplitude sweeps at constant angular frequency of 6.283 rads^{-1} and **(b)** frequency sweep at constant strain of 0.1% of potato protein 'parent' gels at 5 wt% (pink), 10 wt% (blue), 15 wt% (green) and 18 wt% (red) concentration. *G'* (solid symbols) and *G''* (open symbols).

(a)
(b)
(c)

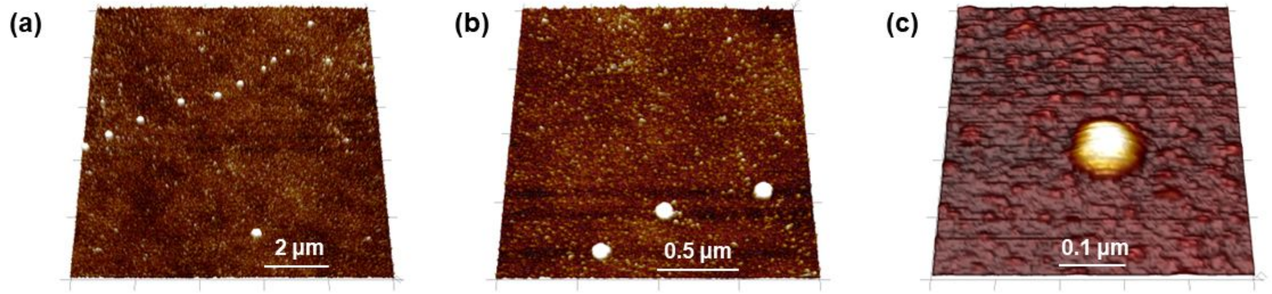

Figure S2. Atomic force microscopy images of potato protein at 1 wt% in fluid (HEPES buffer), illustrating particles in 3-D over a scan area of **(a)** 10 microns **(b)** 2 microns and **(c)** 0.5 micron.

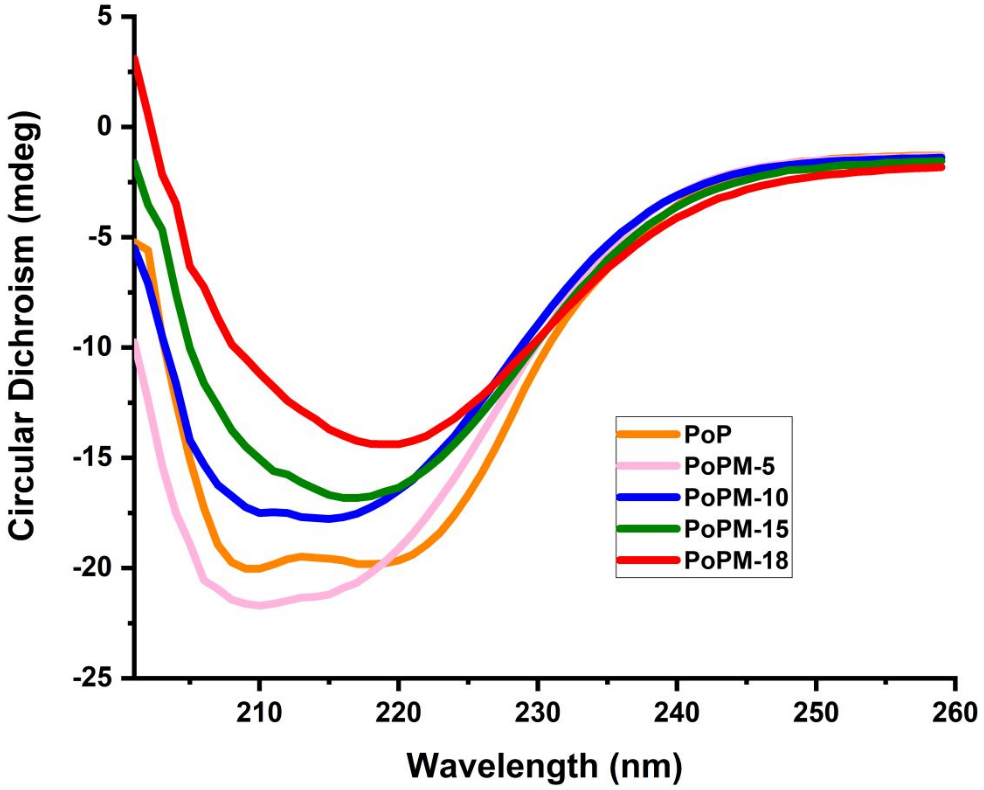

Figure S3. Far UV Spectra for PoPS (orange), PoPM-5 (pink), PoPM-10 (blue), PoPM-15 (green) and PoPM-18 (red) samples.

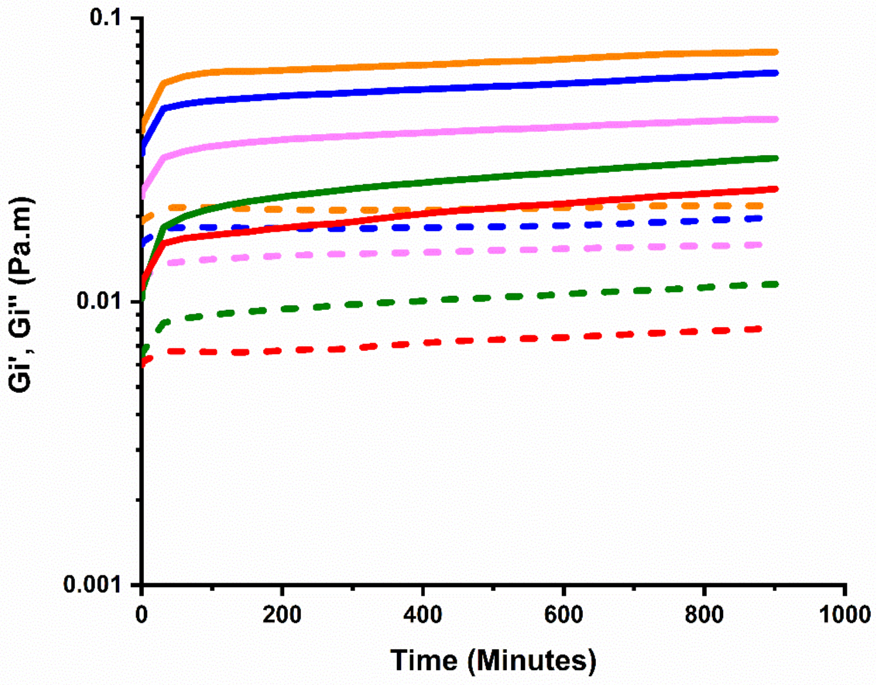

Figure S4. Interfacial shear moduli (G_i' and G_i'') against time at the oil-water interface for 15 h equilibration time, measurements conducted at a constant frequency of 1 Hz. Shown for PoPM samples at 5 wt% (pink), 10 wt% (blue),15 wt% (green) and 18 wt% (red) concentration compared to PoPS (orange).

Table S1. Table displaying the initial decrease in interfacial tension (γ) in comparison to diffusion coefficients (D) and particle size for samples of potato protein (PoPS) and potato protein microgels (PoPM).

Sample	d_H	D ($\mu m^2/s$)	Initial γ decrease (mN.m/s)
PoPS	157.8 nm	2.88	-0.0048 ± 1.2
PoPM-5	93 nm	4.88	-0.0025 ± 7.3
PoPM-10	171.9 nm	2.64	-0.0045 ± 1.6
PoPM-15	253.7 nm	1.79	-0.0060 ± 1.4
PoPM-18	304.9 nm	1.49	-0.0073 ± 1.7

Using the following equation, diffusion coefficients (D) were calculated as D = 454.14/d, where d is particle size and the value of 454.14 was based on a linear fit of values from a study of the diffusion of polystyrene latex microspheres over a similar range of sizes as the protein microgels [1].(A graph of D ($\mu m^2\ s^{-1}$) was plotted against 1/d (μm^{-1}), with the following values extracted from paper [1], 100nm = 4.60 $\mu m^2\ s^{-1}$, 200 nm = 2.15 $\mu m^2\ s^{-1}$, and 1000 nm = 0.472 $\mu m^2\ s^{-1}$, giving a linear fit with slope 454 $\pm$ 9). This was taken as a model system on which to estimate the diffusion of the microgels within our current study. These values were then compared to the decrease in γ for the initial 400 seconds studied, through calculation of the negative gradient via a linear fit of these data.

(1) Shokeen, N.; Issa, C.; Mukhopadhyay, A. Comparison of nanoparticle diffusion using fluorescence correlation spectroscopy and differential dynamic microscopy within concentrated polymer solutions. *Appl. Phys. Lett.* **2017**, *111* (26). DOI: 10.1063/1.5016062.
